# Characterization of WZ4003 and HTH-01-015 as selective inhibitors of the LKB1-tumour-suppressor-activated NUAK kinases

**DOI:** 10.1042/BJ20131152

**Published:** 2013-12-10

**Authors:** Sourav Banerjee, Sara J. Buhrlage, Hai-Tsang Huang, Xianming Deng, Wenjun Zhou, Jinhua Wang, Ryan Traynor, Alan R. Prescott, Dario R. Alessi, Nathanael S. Gray

**Affiliations:** *MRC Protein Phosphorylation and Ubiquitylation Unit, College of Life Sciences, University of Dundee, Dow Street, Dundee DD1 5EH, U.K.; †Department of Cancer Biology, Dana-Farber Cancer Institute, Boston, MA 02115, U.S.A.; ‡Department of Biological Chemistry and Molecular Pharmacology, Harvard Medical School, 250 Longwood Avenue, SGM 628, Boston, MA 02115, U.S.A.; §Division of Cell Signalling and Immunology, College of Life Sciences, University of Dundee, Dow Street, Dundee DD1 5EH, U.K.

**Keywords:** AMP-activated protein kinase (AMPK), AMPK-related kinase 5 (ARK5), kinase inhibitor, kinase profiling, liver kinase B1 (LKB1), myosin phosphate-targeting subunit 1(MYPT1), sucrose-non-fermenting protein kinase/AMPKrelated protein kinase (SNARK), ACC, acetyl-CoA carboxylase, AMPK, AMP-activated protein kinase, BRSK, brain-specific kinase, DMEM, Dulbecco’s modified Eagle’s medium, HA, haemagglutinin, HEK, human embryonic kidney, LKB1, liver kinase B1, MARK, microtubule-affinity-regulating kinase, MEF, mouse embryonic fibroblast, MYPT1, myosin phosphate-targeting subunit 1, NF-κB, nuclear factor κB, PEI, polyethylenimine, PP1, protein phosphatase 1, SIK, salt-induced kinase

## Abstract

The related NUAK1 and NUAK2 are members of the AMPK (AMP-activated protein kinase) family of protein kinases that are activated by the LKB1 (liver kinase B1) tumour suppressor kinase. Recent work suggests they play important roles in regulating key biological processes including Myc-driven tumorigenesis, senescence, cell adhesion and neuronal polarity. In the present paper we describe the first highly specific protein kinase inhibitors of NUAK kinases namely WZ4003 and HTH-01-015. WZ4003 inhibits both NUAK isoforms (IC_50_ for NUAK1 is 20 nM and for NUAK2 is 100 nM), whereas HTH-01-015 inhibits only NUAK1 (IC_50_ is 100 nM). These compounds display extreme selectivity and do not significantly inhibit the activity of 139 other kinases that were tested including ten AMPK family members. In all cell lines tested, WZ4003 and HTH-01-015 inhibit the phosphorylation of the only well-characterized substrate, MYPT1 (myosin phosphate-targeting subunit 1) that is phosphorylated by NUAK1 at Ser^445^. We also identify a mutation (A195T) that does not affect basal NUAK1 activity, but renders it ~50-fold resistant to both WZ4003 and HTH-01-015. Consistent with NUAK1 mediating the phosphorylation of MYPT1 we find that in cells overexpressing drug-resistant NUAK1[A195T], but not wild-type NUAK1, phosphorylation of MYPT1 at Ser^445^ is no longer suppressed by WZ4003 or HTH-01-015. We also demonstrate that administration of WZ4003 and HTH-01-015 to MEFs (mouse embryonic fibroblasts) significantly inhibits migration in a wound-healing assay to a similar extent as NUAK1-knockout. WZ4003 and HTH-01-015 also inhibit proliferation of MEFs to the same extent as NUAK1 knockout and U2OS cells to the same extent as NUAK1 shRNA knockdown. We find that WZ4003 and HTH-01-015 impaired the invasive potential of U2OS cells in a 3D cell invasion assay to the same extent as NUAK1 knockdown. The results of the present study indicate that WZ4003 and HTH-01-015 will serve as useful chemical probes to delineate the biological roles of the NUAK kinases.

## Abstract 2

We describe the discovery of structurally diverse kinase inhibitors to dissect the physiological roles of the NUAK isoforms. We recommend use of an inhibitor-resistant NUAK1[A195T] mutant to verify that the physiological effects of these compounds is indeed mediated through inhibition of NUAKs

## INTRODUCTION

NUAK family SNF1-like kinase-1 [NUAK1, also known as ARK5 (AMPK-related kinase 5)] and the closely related NUAK2 [SNARK (SNF1/AMPK-related kinase)] are members of the AMPK (AMP-activated protein kinase) family of protein kinases that are activated by the LKB1 (liver kinase B1) tumour suppressor protein kinase [1]. NUAK isoforms are ubiquitously expressed and possess an N-terminal kinase domain (residues 55–306, NUAK1), followed by a C-terminal region, which although similar between NUAK1 and NUAK2, possesses no obvious domains or homology with other proteins [2]. LKB1 triggers the activation of NUAK isoforms by phosphorylating a specific threonine residue within their T-loop (Thr^211^ in NUAK1 and Thr^208^ in NUAK2). The other members of the AMPK family activated by LKB1 include AMPKα isoforms, four MARK (microtubule-affinity-regulating kinase) isoforms, three SIK (salt-induced kinase) isoforms, two BRSK (brain-specific kinase)/SAD isoforms and SNRK (SNF-related kinase), a testis-specific enzyme [1,3]. AMPK family kinases play fundamental roles in biology, including regulation of cellular energy levels (AMPKα) [4], cell polarity (MARKs and BRSKs) [5,6], gene transcription (SIKs) [7,8] and resolution of the immune system (SIKs) [8].

There is increasing interest in the NUAK kinases and studies hint at roles in regulating cell adhesion [9,10], cancer cell invasion [11–13], embryonic development [14,15], senescence [16], proliferation [17], neuronal polarity and axon branching [18]. A synthetic lethal siRNA screen also suggested that NUAK1 operates as an essential survival factor in oncogenic Myc-driven tumours and proposed that inhibitors of NUAK1 would have utility for treatment of such tumours [19]. However, despite this flurry of activity, few concrete details are known regarding the key substrates that NUAK isoforms phosphorylate to influence these processes. Only a single substrate, namely the MYPT1 (myosin phosphate-targeting subunit 1) PP1 (protein phosphatase 1) regulatory subunit, has been reported thus far, whose phosphorylation has been demonstrated to be reduced in NUAK1-knockout MEFs (mouse embryonic fibroblasts) [10]. The NUAK1 and NUAK2 isoforms phosphorylate MYPT1 at three conserved residues (Ser^445^, Ser^472^ and Ser^910^) following conditions that induce cell detachment [10]. This promotes interaction with 14-3-3 isoforms, interfering with the ability of the MYPT1–PP1 phosphatase complex to dephosphorylate the myosin light chain [10]. Both isoforms of NUAK possess three unique GILK motifs that interact with PP1, and this interaction is essential for association of NUAK isoforms with MYPT1 [10]. It is likely that both NUAK1 and NUAK2 isoforms phosphorylate MYPT1 at Ser^445^ and that the residual phosphorylation of MYPT1 observed in NUAK1-knockout MEFs is mediated by NUAK2 [10].

In overexpression and *in vitro* studies, given the similarity in the catalytic domains of AMPK family kinases, it is likely that these kinases will phosphorylate non-physiological substrates normally phosphorylated by other family members. To avoid having to rely on *in vitro* and overexpression approaches, efforts have commenced to develop selective AMPK family kinase inhibitors. Early AMPK family inhibitors such as Compound C (also known as dorsomorphin) [20] and BX-795 [10,19,21] inhibited all of the AMPK family members tested, including NUAK isoforms, with high potency. Subsequently, a BX-795 derivative termed MRT67307 was described that exhibited greater specificity, but nevertheless still inhibited SIK, NUAK and MARK isoforms [22]. However, the recent discovery of two small molecules termed KIN112 and HG-9-91-01 [8,23] that inhibit all three SIK isoforms without significantly suppressing other AMPK family kinases, offers encouragement that it will be feasible to develop specific AMPK family inhibitors. In the present paper we provide further evidence that this is indeed the case. We report on two highly selective inhibitors termed WZ4003, which inhibits both NUAK1 and NUAK2, and HTH-01-015, which inhibits NUAK1 with >100-fold higher potency than NUAK2. We show that WZ4003 and HTH-01-015 are capable of suppressing MYPT1 phosphorylation in cells and phenocopy knock out of NUAK1 in cell migration and adhesion analyses. The results of the present study establish that HTH-01-015 and WZ4003 comprise useful tools for probing the physiological functions of the NUAK isoforms.

## MATERIALS AND METHODS

### Materials

The Sakamototide substrate peptide (ALNRTSSDSALHRRR) was used as the NUAK1 and NUAK2 substrate in kinase assays [10]. [γ-^32^P]ATP was from PerkinElmer. Protein G–Sepharose, glutathione–Sepharose and an ECL kit was from GE Healthcare. P81 phosphocellulose paper was from Whatman. Doxycycline, DMSO, BSA and benzamidine were from Sigma–Aldrich. PMSF was from Melford. Novex 4–12% polyacrylamide Bis-Tris gels, LDS sample buffer, puromycin, hygromycin, blasticidin, PBS-EDTA-based Cell Dissociation Buffer and other tissue culture reagents were from Invitrogen Life Technologies. Instant Blue Coomassie stain was from Expedeon. PEI (polyethylenimine) was from Polysciences, and 1 M magnesium acetate solution was from Fluka.

### Antibodies

The following antibodies were raised in sheep and affinity-purified on the appropriate antigen: anti-(MYPT1 p-Ser^445^) (residues 437–452 of mouse, sequence RLGLRKTGS*YGALAEI, S508C, first bleed), anti-MYPT1 [human MBP (maltose-binding protein)–MYPT1, residues 714–1005, S662B, first bleed] and anti-NUAK1 (human His–NUAK1, S628B, second bleed). Antibody production was carried out under UK Home Office approved guidelines. The commercial antibodies used in the present paper are anti-ACC (acetyl-CoA carboxylase) (Cell Signaling Technology, catalogue number 3662), anti-(ACC p-Ser^79^) (Cell Signaling Technology, catalogue number 3661), anti-HA (haemagglutinin)–peroxidase (3F10) (Roche, catalogue number 12013819001) and all HRP (horseradish peroxidase)-conjugated secondary antibodies were obtained from Thermo Scientific.

### General methods

All recombinant DNA procedures, electrophoresis, immunoblotting, immunoprecipitation and tissue culture were performed using standard protocols. NUAK1[A195T] mutagenesis was performed using the QuikChange® site-directed mutagenesis method (Stratagene) with KOD polymerase (Novagen). DNA constructs used for transfection were purified from *Escherichia coli* DH5α using Qiagen Maxi-prep kits according to the manufacturer's protocol. All DNA constructs were verified by DNA sequencing, which was performed by the Sequencing Service (MRC Protein Phosphorylation Unit, College of Life Sciences, University of Dundee, Dundee, U.K.; http://www.dnaseq.co.uk), using DYEnamic ET terminator chemistry (GE Healthcare) on Applied Biosystems automated DNA sequencers.

### Cell culture, treatments and cell lysis

HEK (human embryonic kidney)-293 and U2OS cells were cultured in DMEM (Dulbecco's modified Eagle's medium) supplemented with 10% FBS, 2 mM glutamine and 1×antibacterial/antimycotic solution. NUAK1^+/+^ and NUAK1^−/−^ MEFs were cultured in DMEM supplemented with 10% (v/v) FBS and 2 mM glutamine, 1×antibacterial/antimycotic solution, 1% (v/v) non-essential amino acids and 1% (v/v) sodium pyruvate. HEK-293 Flp/In T-Rex cell lines were cultured in DMEM supplemented with 10% (v/v) FBS and 2 mM glutamine, 1×antibacterial/antimycotic solution, 100 μg/ml hygromycin and 15 μg/ml blasticidin. Supplementing the culture medium with 0.1 μg/ml doxycycline for 16–24 h induced protein expression in the HEK-293 Flp/In T-Rex cells. Cell counting was carried out using Invitrogen Countess following the manufacturer's protocol. A cell-detachment assay was carried out on HEK-293 cells using PBS-EDTA-based cell dissociation buffer as described previously [10]. An inhibitor dose-dependence assay was carried out by treating the cells with various concentrations of the inhibitors as indicated in the Figure legends. The inhibitors were dissolved in DMSO and the total concentration of DMSO in the culture media never exceeded 1%. Transient transfections of HEK-293 cells were carried out using PEI [24]. Stable transfections were carried out in HEK-293 Flp/In T-Rex cells (Invitrogen) following the manufacturer's protocol. Lentivirus-mediated knock down of NUAK1 was carried out in U2OS cells using shRNA constructs as described previously [10]. Post-treatment and/or transfection, cells were lysed in lysis buffer containing 50 mM Tris/HCl (pH 7.5), 1 mM EGTA, 1 mM EDTA, 1% Triton X-100, 50 mM NaF, 10 mM sodium 2-glycerophosphate, 5 mM sodium pyrophosphate, 1 mM sodium orthovanadate, 0.27 M sucrose, 1 mM benzamidine (added before lysis), 1 mM PMSF (added before lysis) and 0.1% 2-mercaptoethanol (added before lysis). Lysates were clarified by centrifugation at 16000 ***g*** for 15 min at 4°C and either used for further experiments or snap-frozen in liquid nitrogen and stored at −80°C. Protein estimation was carried out using the Bradford method with BSA as a standard.

### IC_50_ determination

Active GST–NUAK1, GST–NUAK1[A195T] and GST–NUAK2 enzymes were purified using glutathione–Sepharose from HEK-293 cell lysates 36–48 h following the transient transfection of pEBG2T mammalian constructs expressing N-terminal GST-tagged NUAK1, NUAK1[A195T] or NUAK2. For peptide kinase assays, 96-well plates were used, and each reaction was performed in triplicate. Each reaction was set up in a total volume of 50 μl containing 100 ng of NUAK1 (wild-type or A195T mutant) or NUAK2 in 50 mM Tris/HCl (pH 7.5), 0.1 mM EGTA, 10 mM magnesium acetate, 200 μM Sakamototide, 0.1 mM [γ-^32^P]ATP (450–500 c.p.m./pmol) and the indicated concentrations of inhibitors dissolved in DMSO. After incubation for 30 min at 30°C, reactions were terminated by adding 25 mM (final) EDTA to chelate the magnesium. Then, 40 μl of the reaction mix was spotted on to P81 paper and immersed in 50 mM orthophosphoric acid. Samples were washed three times in 50 mM orthophosphoric acid followed by a single acetone rinse and air drying. The incorporation of [γ-^32^P]ATP into Sakamototide was quantified by Cerenkov counting. The values were expressed as a percentage of the DMSO control. IC_50_ curves were developed and IC_50_ values were calculated using GraphPad Prism software.

### Kinase activity assays

*In vitro* activities of purified GST–NUAK1 and GST–NUAK1[A195T] were measured using Cerenkov counting of incorporation of radioactive ^32^P from [γ-^32^P]ATP into Sakamototide substrate peptide as described previously [10]. Reactions were carried out in a 50 μl reaction volume for 30 min at 30°C and reactions were terminated by spotting 40 μl of the reaction mix on to P81 paper and immediately immersing in 50 mM orthophosphoric acid. Samples were washed three times in 50 mM orthophosphoric acid followed by a single acetone rinse and air drying. The kinase-mediated incorporation of [γ-^32^P]ATP into Sakamototide was quantified by Cerenkov counting. One unit of activity was defined as that which catalysed the incorporation of 1 nmol of [^32^P]phosphate into the substrate over 1 h.

### Wound-healing assay

MEFs were split and an approximately equal number of cells were loaded into the left and right chambers of the IBIDI Self-Insertion Inserts (catalogue number 80209). Each insert was placed in one well of a 12-well plate and the cells were seeded with or without treatment with the inhibitors. For the comparison of the migration properties of different MEFs on the same video, a single insert was used and an equal number of MEFs were counted and loaded on either chamber of the same insert. To study the effect of inhibitors on cell migration, wound-healing assays on MEFs were also carried out on separate inserts with or without treatment with a 10 μM concentration of WZ4003 or HTH-01-015. Inhibitors were added to the cells 1 h before the start of the migration assay. The experiments were carried out in triplicate. After overnight incubation at 37°C and 5% CO_2_, the insert was removed and the migration of cells into the 500 μm gap between the chambers was observed. The wound-gap healing properties of the cells were observed over a period of 15–20 h under a Nikon Eclipse Ti microscope with images taken every 2 min by a Photometrics cascade II CCD (charge-coupled device) camera using Nikon NIS Elements software.

### Cell proliferation assay

Cell proliferation assays were carried out colorimetrically in 96-well plates using the CellTiter 96® AQueous Non-Radioactive Cell Proliferation Assay kit (Promega) following the manufacturer's protocol. Initially, 2000 cells per well were seeded for U2OS cells and 3000 cells per well were seeded for MEFs. The proliferation assays were carried out over 5 days in the presence or absence of 10 μM HTH-01-015 or WZ4003.

### Cell invasion assay

The ability of U2OS cells to invade in the presence or absence of 10 μM HTH-01-015 or WZ4003 was tested in a growth-factor-reduced Matrigel™ invasion chamber (BD Biosciences, catalogue number 354483) as described previously [25]. Cells were serum-deprived for 2 h, detached using cell-dissociation buffer (Gibco), and 2.5×10^5^ cells suspended in DMEM containing 1% (w/v) BSA were added to the upper chambers in triplicate and chemoattractant [DMEM containing 10% (v/v) FBS] was added to the lower wells. The chambers were kept at 37°C in 5% CO_2_ for 16 h in the presence or absence of 10 μM HTH-01-015 or WZ4003 both in the upper and lower wells. Non-invaded cells were removed from the upper face of the filters by scraping, and cells that had migrated to the lower face of the filters were fixed and stained with Reastain Quick-Diff kit (Reagena) and images (×10 magnification) were captured. For cell invasion assays, statistical significance was assessed using GraphPad Prism 5.0.

### Protein kinase profiling

Kinase inhibitor specificity profiling assays were carried out at The International Centre for Protein Kinase Profiling (http://www.kinase-screen.mrc.ac.uk/) against a panel of 140 protein kinases as described previously [26,27]. Results are presented as a percentage of kinase activity in DMSO control reactions. Protein kinases were assayed *in vitro* with 0.1 or 1 μM of the inhibitors and the results are presented as an average of triplicate reactions±S.D. or in the form of comparative histograms.

## RESULTS

### WZ4003 is a dual inhibitor of NUAK1 and NUAK2

The NUAK inhibitors reported in the present paper were obtained by re-purposing, in the case of WZ4003, or re-optimization, in the case of HTH-01-015, of known compounds. The report that BX795 [28], a tri-substituted pyrimidine whose primary targets are TBK1 {TANK [TRAF (tumour-necrosis-factor-receptor-associated factor)-associated NF-κB (nuclear factor-κB) activator]-binding kinase 1} and IKKϵ [IκB (inhibitor of NF-κB) kinase ϵ], inhibits NUAK1 [21,29] inspired us to evaluate NUAK inhibitory activity of our collection of 2,4,5-tri-substituted pyrimidines. WZ4003 was identified from this effort [30]. HTH-01-015 was derived from LRRK2-IN [31] and related pyrimido-diazepines previously reported by us to have a weak inhibitory effect against NUAK1 [32]. The chemical synthesis schemes used to generate and characterize each of the compounds used in the present study is detailed in the Supplementary Online Data (at http://www.biochemj.org/bj/457/bj4570215add.htm).

**Figure 1:**
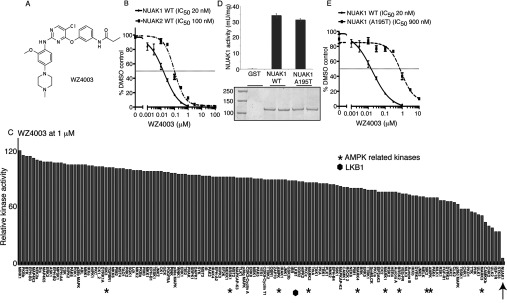
WZ4003, a specific NUAK1 and NUAK2 inhibitor (**A**) Chemical structure of the NUAK1/NUAK2 inhibitor WZ4003. (**B**) Wild-type (WT) GST–NUAK1 and GST–NUAK2 were assayed using 200 μM Sakamototide in the presence of 100 μM [γ-^32^P]ATP (~500 c.p.m./pmol) with the indicated concentrations of WZ4003. The IC_50_ graph was plotted using GraphPad Prism software with non-linear regression analysis. The results are presented as the percentage of kinase activity relative to the DMSO-treated control. Results are means±S.D. for triplicate reactions with similar results obtained in at least one other experiment. (**C**) Kinase profiling of the WZ4003 inhibitor at 1 μM was carried out against the panel of 140 kinases at the The International Centre for Protein Kinase Profiling (http://www.kinase-screen.mrc.ac.uk/). AMPK family kinases are indicated with an asterisk, LKB1 with a filled hexagon and NUAK1 with an arrow. The full names of the kinases can be found in the legend to Supplementary Table S1 (at http://www.biochemj.org/bj/457/bj4570215add.htm). (**D**) Wild-type (WT) GST–NUAK1 and GST–NUAK1[A195T] were purified from HEK-293 cells following transient transfection and relative levels of wild-type and mutant enzymes were analysed by Coomassie Blue staining of a polyacrylamide gel (bottom panel). Intrinsic kinase activities of the equivalent amounts of NUAK1 and NUAK1[A195T] were compared by carrying out a quantitative kinase activity assay by calculating the relative kinase-mediated incorporation of [γ-^32^P]ATP into the Sakamototide substrate peptide. Values are means±S.D. for an experiment carried out in triplicate. (**E**) As in (**B**) except that WZ4003 comparative IC_50_ values were derived for wild-type (WT) GST–NUAK1 and GST—NUAK1[A195T].

The structure of WZ4003 is shown in Figure 1(A). It inhibits NUAK1 with an IC_50_ of 20 nM (Figure 1B) and NUAK2 with an IC_50_ of 100 nM (Figure 1B). To evaluate the specificity of WZ4003 we studied the effect that this compound has on the activity of 140 protein kinases, including ten AMPK-related kinase family members most closely related to NUAK1 (Figure 1C and Supplementary Table S1 at http://www.biochemj.org/bj/457/bj4570215add.htm). WZ4003 was remarkably specific and, apart from NUAK1 and NUAK2, did not significantly inhibit ten other AMPK-related kinases or other kinases tested, including LKB1 at a concentration of 1 μM (10-fold higher than the IC_50_ of inhibition of NUAK1).

### HTH-01-015 is a selective inhibitor of NUAK1

**Figure 2:**
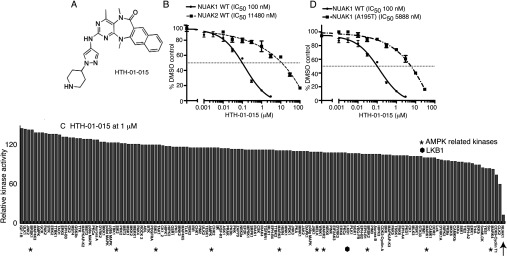
HTH-01-015, a specific NUAK1 inhibitor (**A**) Chemical structure of the NUAK1-specific inhibitor HTH-01-015. (**B**) Wild-type (WT) GST–NUAK1 and GST–NUAK2 were assayed using 200 μM Sakamototide in the presence of 100 μM [γ-^32^P]ATP (~500 c.p.m./pmol) with the indicated concentrations of HTH-01-015. The IC_50_ graph was plotted using Graphpad Prism software with non-linear regression analysis. The results are presented as the percentage of kinase activity relative to the DMSO-treated control. Results are means±S.D. for triplicate reactions with similar results obtained in at least one other experiment. (**C**) Kinase profiling of the HTH-01-015 inhibitor at 1 μM was carried out against the panel of 140 kinases at the The International Centre for Protein Kinase Profiling (http://www.kinase-screen.mrc.ac.uk/). AMPK family kinases are indicated with an asterisk, LKB1 with a filled hexagon and NUAK1 with an arrow. The full names of the kinases can be found in the legend to Supplementary Table S1 (at http://www.biochemj.org/bj/457/bj4570215add.htm). (**D**) As in (**B**) except that HTH-01-015 comparative IC_50_ values were derived for wild-type (WT) GST–NUAK1 and GST–NUAK1[A195T].

The structure of HTH-01-015 is shown in Figure 2(A). It inhibits NUAK1 with an IC_50_ of 100 nM (Figure 2B), but, unlike WZ4003, does not significantly inhibit NUAK2 (IC_50_ of >10 μM) (Figure 2B). HTH-01-015 was similarly specific to WZ4003 and, apart from NUAK1, did not markedly suppress the activity of any of the other 139 protein kinases evaluated (Figure 2C and Supplementary Table S1).

**Figure 3:**
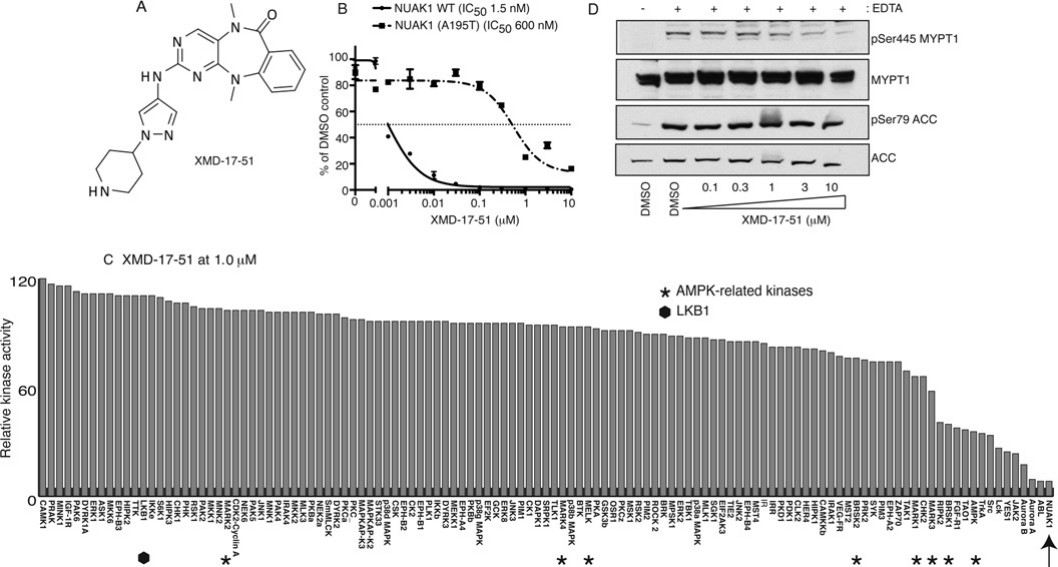
XMD-17-51, a potent semi-specific NUAK1 inhibitor (**A**) Chemical structure of XMD-17-51. (**B**) Wild-type (WT) GST–NUAK1 and GST–NUAK1[A195T] were assayed using 200 μM Sakamototide in the presence of 100 μM [γ-^32^P]ATP (~500 c.p.m./pmol) with the indicated concentrations of XMD-17-51. The IC_50_ graph was plotted using Graphpad Prism software with non-linear regression analysis. The results are presented as the percentage of kinase activity relative to the DMSO-treated control. Results are means±S.D. for triplicate reactions with similar results obtained in at least one other experiment. (**C**) Kinase profiling of the XMD-17-51 inhibitor at 1 μM was carried out against the panel of 140 kinases at the The International Centre for Protein Kinase Profiling (http://www.kinase-screen.mrc.ac.uk/). AMPK family kinases are indicated with an asterisk, LKB1 with a filled hexagon and NUAK1 with an arrow. The full names of the kinases can be found in the legend to Supplementary Table S1 (at http://www.biochemj.org/bj/457/bj4570215add.htm). (**D**) HEK-293 cells were treated in the absence (DMSO) or presence of the indicated concentrations of XMD-17-51 over 16 h. Cell medium was then replaced with either normal DMEM containing no EDTA-PBS-based cell dissociation buffer (−) or EDTA-PBS-based cell dissociation buffer (+) containing the same concentration of XMD-17-51 that the cells were previously incubated in. Cell detachment was induced with gentle tapping of the plates followed by gentle centrifugation at 70 ***g*** for 3 min. Cells were lysed immediately after removal of the supernatant. Endogenous MYPT1 was immunoprecipitated from 0.5 mg of the cell lysates. The immunoprecipitates were immunoblotted for the detection of p-Ser^445^ MYPT1 and total MYPT1. The cell lysates were subjected to immunoblotting for the detection of p-Ser^79^ ACC and total ACC. Similar results were obtained in three separate experiments.

**Figure 4:**
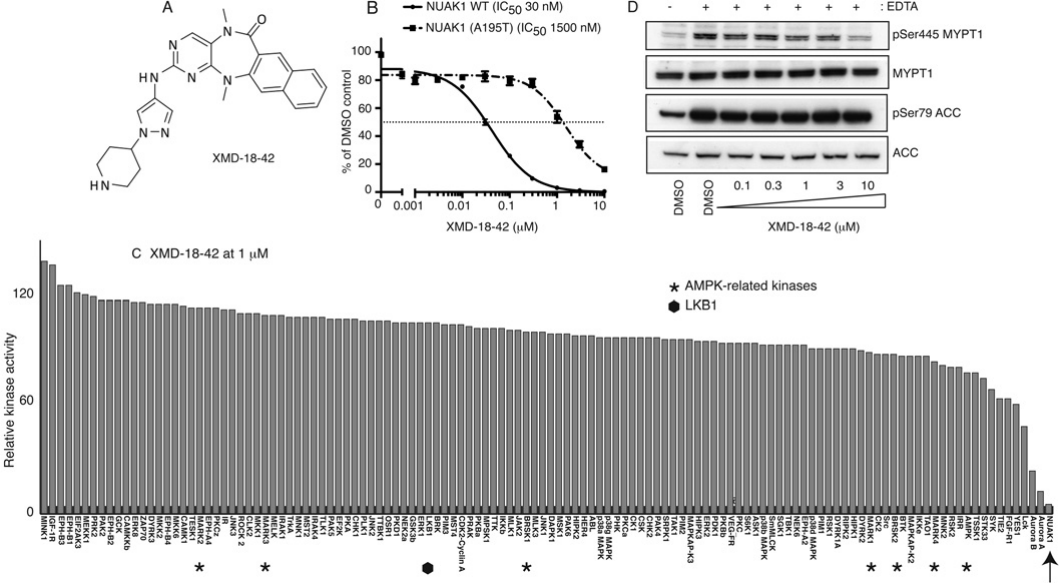
XMD-18-42, a semi-specific NUAK1 inhibitor (**A**) Chemical structure of XMD-18-42. (**B**) Wild-type (WT) GST–NUAK1 and GST–NUAK1[A195T] were assayed using 200 μM Sakamototide in the presence of 100 μM [γ-^32^P]ATP (~500 c.p.m./pmol) with the indicated concentrations of XMD-18-42. The IC_50_ graph was plotted using Graphpad Prism software with non-linear regression analysis. The results are presented as the percentage of kinase activity relative to the DMSO-treated control. Results are means±S.D. for triplicate reactions with similar results obtained in at least one other experiment. (**C**) Kinase profiling of the XMD-18-42 inhibitor at 1 μM was carried out against the panel of 140 kinases at the The International Centre for Protein Kinase Profiling (http://www.kinase-screen.mrc.ac.uk/). AMPK family kinases are indicated with an asterisk, LKB1 with a filled hexagon and NUAK1 with an arrow. The full names of the kinases can be found in the legend to Supplementary Table S1 (at http://www.biochemj.org/bj/457/bj4570215add.htm). (**D**) HEK-293 cells were treated in the absence (DMSO) or presence of the indicated concentrations of XMD-18-42 over 16 h. Cell medium was then replaced with either normal DMEM containing no EDTA-PBS-based cell dissociation buffer (−) or EDTA-PBS-based cell dissociation buffer (+) containing the same concentration of XMD-18-42 that the cells were previously incubated in. Cell detachment was induced with gentle tapping of the plates followed by gentle centrifugation at 70 ***g*** for 3 min. Cells were lysed immediately after removal of the supernatant. Endogenous MYPT1 was immunoprecipitated from 0.5 mg of the cell lysates. The immunoprecipitates were immunoblotted for the detection of p-Ser^445^ MYPT1 and total MYPT1. The cell lysates were subjected to immunoblotting for the detection of p-Ser^79^ ACC and total ACC. Similar results were obtained in three separate experiments.

We also generated two further analogues of HTH-01-015, namely XMD-17-51 (Figure 3A) and XMD-18-42 (Figure 4A), that inhibited NUAK1 more potently than HTH-01-015. XMD-17-51 inhibited NUAK1 with an IC_50_ of 1.5 nM (Figure 3B) and XMD-18-42 inhibited NUAK1 with an IC_50_ of 30 nM (Figure 4B). Neither compound significantly inhibited NUAK2 (results not shown). However, XMD-17-51 and XMD-18-42 were less selective than WZ4004 and HTH-01-015 and inhibited kinases involved in growth and proliferation, such as Aurora isoforms, ABL (Abelson tyrosine-protein kinase 1) and JAK2 (Janus kinase 2) (Figures 3C and 4C). XMD-17-51 also inhibited several AMPK family members (MARK1, MARK3, BRSK1 and AMPK) (Figure 3C).

### Development of inhibitor-resistant NUAK1 mutants

Previous work revealed that in other kinases, such as PKA (cAMP-dependent protein kinase) [33], ROCK (Rho-associated kinase) [33] and LRRK2 (leucine-rich repeat kinase 2) [31,34], mutation of the alanine residue that resides before the conserved subdomain VII magnesium ion-binding DFG motif to a threonine residue, introduces a steric clash with certain ATP-competitive inhibitors without affecting the intrinsic specific kinase activity. As NUAK isoforms also possess an alanine residue at the equivalent position (Ala^195^), we mutated this residue to a threonine residue. Importantly, this mutation did not inhibit NUAK1 specific activity (Figure 1D), but markedly reduced the potency of WZ4003 (45-fold, Figure 1E) and HTH-01-015 (~60-fold, Figure 2D). The A195T mutation also rendered NUAK1 >50-fold resistant to the more potent, but less selective, XMD-17-51 (Figure 3C) and XMD-18-42 (Figure 4C) NUAK1 inhibitors.

### WZ4003 and HTH-01-015 suppress NUAK1-mediated MYPT1 phosphorylation

**Figure 5:**
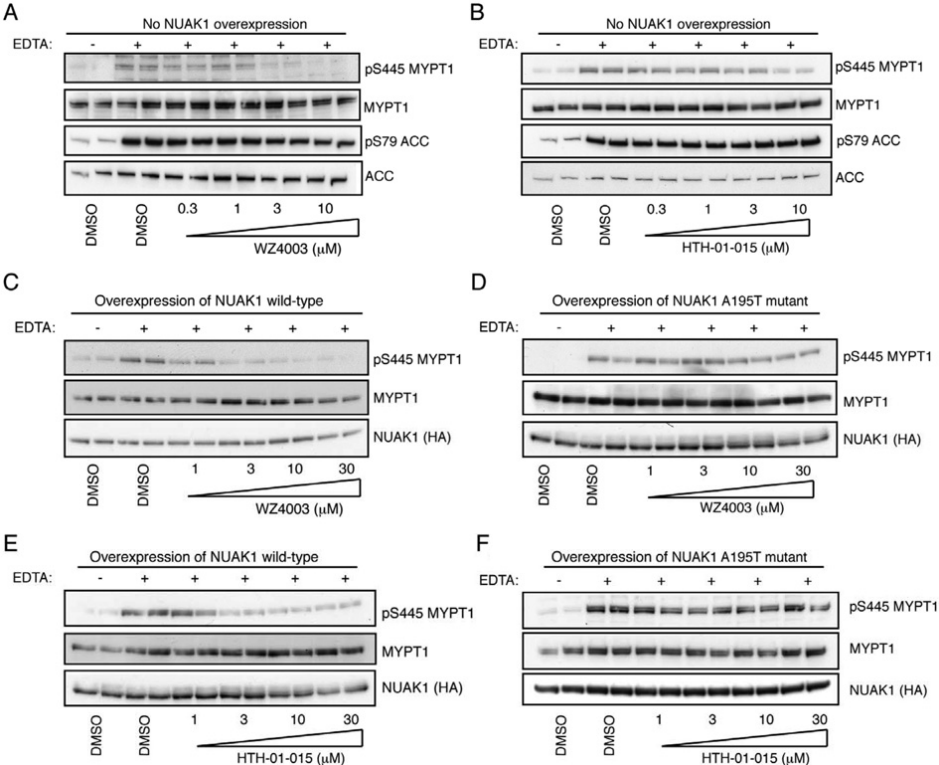
HTH-01-015 and WZ4003 inhibit MYPT1 Ser^445^ phosphorylation *in vivo* (**A**) HEK-293 cells were treated in the absence (DMSO) or presence of the indicated concentrations of WZ4003 over 16 h. Cell medium was then replaced with either normal DMEM containing no EDTA-PBS-based cell dissociation buffer (−) or EDTA-PBS-based cell dissociation buffer (+) containing the same concentration of WZ4003 that the cells were previously incubated in. Cell detachment was induced with gentle tapping of the plates followed by gentle centrifugation at 70 ***g*** for 3 min. Cells were lysed immediately after removal of the supernatant. Endogenous MYPT1 was immunoprecipitated from 0.5 mg of the cell lysates. The immunoprecipitates were immunoblotted for the detection of p-Ser^445^ MYPT1 and total MYPT1. The cell lysates were subjected to immunoblotting for the detection of p-Ser^79^ ACC and total ACC. Similar results were obtained in three separate experiments. (**B**) As in (**A**) except for the HTH-01-015 inhibitor was used. (**C**–**F**) As above except that HEK-293 Flp/In T-Rex cells stably expressing the indicated wild-type HA-tagged NUAK1 or drug-resistant HA-tagged NUAK1[A195T] were used. Similar results were obtained in three separate experiments for all data shown on this Figure.

To evaluate whether WZ4003 and HTH-01-015 could suppress NUAK activity *in vivo*, we treated HEK-293 cells with increasing concentrations of either inhibitor and assessed its effect on MYPT1 phosphorylation at Ser^445^, one of the major sites of NUAK1 phosphorylation [10]. We treated HEK-293 cells with EDTA to induce detachment and phosphorylation of Ser^445^ [10], and observed that WZ4003 suppressed MYPT1 phosphorylation in a dose-dependent manner, with maximal effects observed at inhibitor concentrations of 3–10 μM (Figure 5A). As HEK-293 cells express NUAK1 as well as NUAK2, and previous work suggests that both of these kinases interact and phosphorylate MYPT1 [10], it is likely that a NUAK1-selective inhibitor would not suppress MYPT1 phosphorylation to the same extent as the dual NUAK isoform inhibitor. Consistent with this we found that treatment of cells with 10 μM HTH-01-015, the NUAK1 isoform selective inhibitor, only led to a partial inhibition of MYPT1 phosphorylation (Figure 5B). The other compounds, XMD-17-51 (Figure 3D) and XMD-18-42 (Figure 4D), that potently inhibit NUAK1 but not NUAK2, also only partially suppressed MYPT1 phosphorylation.

EDTA-triggered cell detachment also potently activates AMPK [10] and therefore induces phosphorylation of one of its substrates, ACC, at Ser^79^ [35]. Consistent with the screening data indicating that WZ4003 and HTH-01-015 do not inhibit AMPK, we observed that neither compound inhibited phosphorylation of ACC at Ser^79^ induced by cell detachment (Figures 5A and 5B).

To obtain further evidence that the WZ4003 and HTH-01-015 compounds inhibited NUAK activity *in vivo*, we generated HEK-293 cells that stably overexpress inhibitor-sensitive wild-type HA–NUAK1 or inhibitor-resistant HA–NUAK1[A195T] cells. Quantitative immunoblot analysis revealed that the wild-type and mutant NUAK1 were expressed ~150-fold and ~75-fold higher respectively than endogenous NUAK1 (Supplementary Figure S1 at http://www.biochemj.org/bj/457/bj4570215add.htm). Strikingly, in cells expressing drug-resistant NUAK1[A195T], we observed that even at very high concentrations of 30 μM, WZ4003 (Figure 5D) or HTH-01-015 (Figure 5F) failed to block MYPT1 Ser^445^ phosphorylation. In contrast, in HEK-293 cells expressing wild-type NUAK1, concentrations of 3–10 μM WZ4003 (Figure 5C) or HTH-01-015 (Figure 5E) markedly suppressed phosphorylation of MYPT1.

### WZ4003 and HTH-01-015 suppresses cell migration

**Figure 6:**
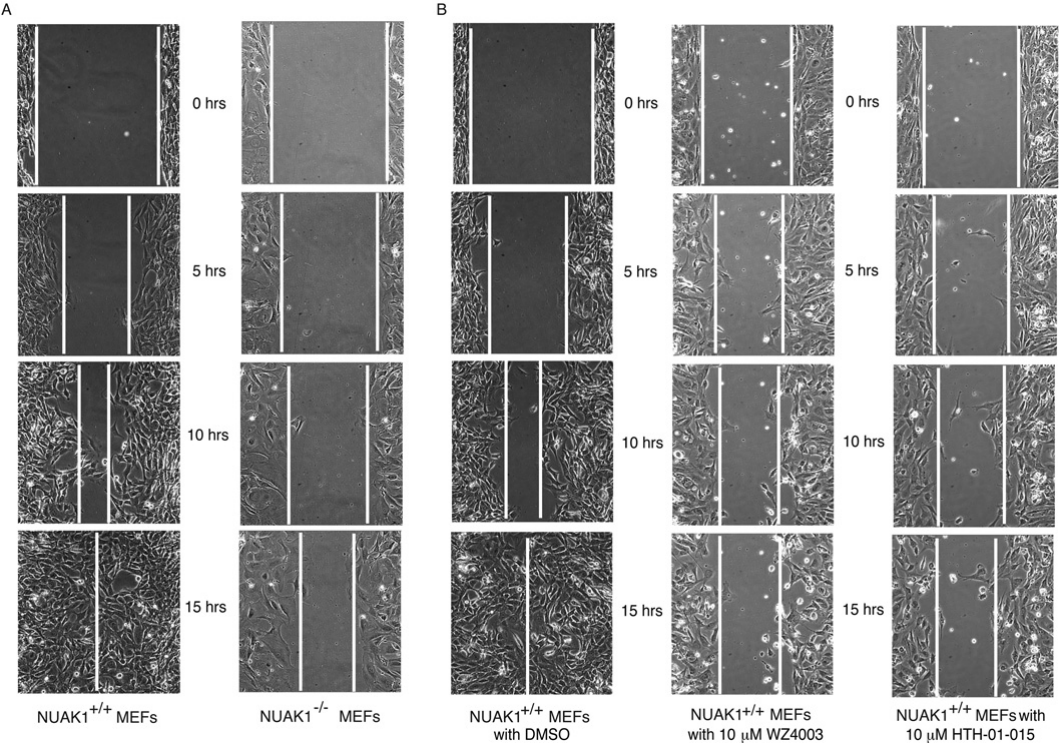
NUAK1 inhibition suppresses cell migration (**A**) NUAK1^+/+^ and NUAK1^−/−^ MEFs were split into the chambers (as described in the Materials and methods section). The inserts were then removed and a wound-healing assay was carried out in triplicate. Snapshots at specific time points from time-lapse microscopy were used as representative images for comparison between the migration properties of NUAK1^+/+^ and NUAK1^−/−^ MEFs. (**B**) The migration assay of NUAK1^+/+^ MEFs treated with or without 10 μM WZ4003 or HTH-01-015 was carried out as in (**A**).

Previous work suggested that RNAi-mediated knock down of NUAK1 promoted cell adhesion [10], which would be expected to inhibit cell migration. To investigate this further with a view to assessing whether NUAK inhibitors would inhibit migration, we first compared the migration of wild-type (NUAK1^+/+^) and homozygous NUAK1-knockout (NUAK1^−/−^) MEFs using a 2D wound-healing assay. Consistent with NUAK1^−/−^ MEFs being more adhesive, we found that they migrated slower than wild-type cells and presented a more ‘flattened’ adherent phenotype (Figure 6A). A movie comparing migration of the NUAK1^+/+^ and NUAK1^−/−^ MEFs also highlights the strikingly reduced motility and more compressed phenotype of the NUAK1^−/−^ MEFs (Supplementary Movie S1 at http://www.biochemj.org/bj/457/bj4570215add.htm). This phenotype could be largely rescued by retroviral overexpression of NUAK1^+/+^ into NUAK1^−/−^ MEFs (Supplementary Movie S2 at http://www.biochemj.org/bj/457/bj4570215add.htm). We next investigated whether the WZ4003 and HTH-01-015 inhibitors could inhibit cell migration and observed that treatment of NUAK1^+/+^ MEFs with 10 μM WZ4003 or HTH-01-015 markedly reduced cell migration in the wound-healing assay (Figure 6B).

### WZ4003 and HTH-01-015 inhibit cell proliferation

**Figure 7:**
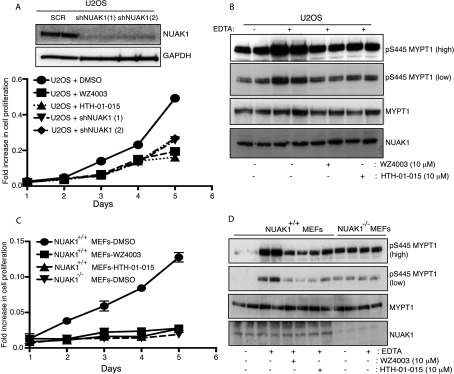
NUAK1 inhibition suppresses cell proliferation (**A**) U2OS cells were incubated with or without 10 μM WZ4003 or 10 μM HTH-01-015 and a cell proliferation assay was carried out over 5 days in triplicate using the CellTiter 96® AQueous Non-Radioactive Cell Proliferation Assay kit (Promega) (as described in the Materials and methods section). U2OS cells in which NUAK1 has been knocked-down using two different shRNA hairpins were used in parallel as controls. The efficiency of the knock down of each shRNA is shown in top panel. SCR, control scrambled shRNA hairpin; shNUAK1 (1), first NUAK1 shRNA hairpin; shNUAK1 (2), second NUAK1 shRNA hairpin. (**B**) U2OS cells were treated with (+) or without (−) 10 μM WZ4003 or 10 μM HTH-01-015. After 16 h cell media was removed and cells were treated with EDTA-PBS-based cell dissociation buffer supplemented with 10 μM WZ4003, 10 μM HTH-01–015 or DMSO for 20 min. Cell detachment was induced with gentle tapping of the plates followed by gentle centrifugation at 70 ***g*** for 3 min. Cells were lysed immediately after removal of the media and immunoblotted for the detection of the indicated antibodies. (**C** and **D**) As above, except NUAK1^+/+^ and NUAK1^−/−^ MEFs were used. Similar results were obtained in three separate experiments.

Previous studies have suggested that inhibiting NUAK1 would suppress proliferation [17]. We therefore checked whether NUAK1 inhibition by 10 μM WZ4003 or HTH-01-015 impaired the proliferation of U2OS cells (Figures 7A and 7B) or MEFs (Figures 7C and 7D). In U2OS cells we found that either inhibitor suppressed proliferation (Figure 7A) and phosphorylation of MYPT1 (Figure 7B) to the same extent as shRNA-mediated NUAK1 knockdown. In MEFs we also observed that treatment with 10 μM WZ4003 or HTH-01-015 suppressed proliferation (Figure 7C) and phosphorylation of MYPT1 (Figure 7D) to the same extent as NUAK1-knockout.

### WZ4003 and HTH-01-015 inhibit U2OS cell invasion

**Figure 8:**
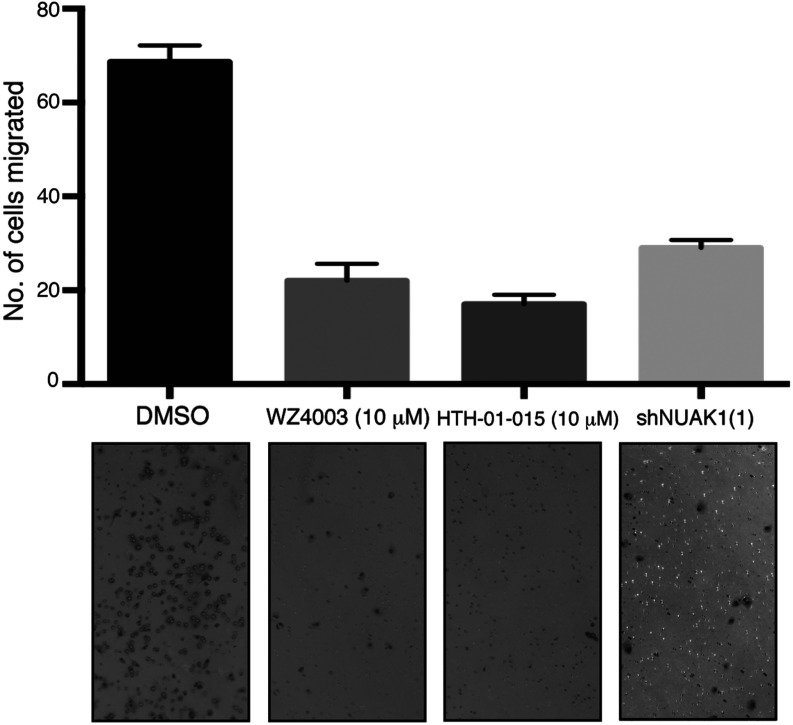
NUAK1 inhibition suppresses invasion potential U2OS cells treated without (DMSO) or with 10 μM WZ4003 or 10 μM HTH-01-015 were plated on to a Transwell® invasion assay plate in triplicate using 10% FBS as a chemoattractant. Cells that had invaded through to the lower face of the filters were fixed and photographed (×10 magnification). The cells that migrated were counted and the data are presented as the mean number of migrated cells±S.D. U2OS cells with NUAK1-knockdown were used in parallel as a control. Similar results were obtained in two separate experiments with each condition analysed in triplicate.

Previous work has implicated NUAK1 in controlling the invasive ability of various cell types [11–13]. To test whether NUAK1 inhibition impaired the ability of the invasive U2OS cells to enter a matrix, we used a 3D Matrigel™ Transwell® invasion assay [36]. These assays demonstrated that 10 μM WZ4003 or HTH-01-015 markedly inhibited the invasiveness of U2OS cells in this assay (Figure 8).

## DISCUSSION

WZ4003 and HTH-01-015 are remarkably selective NUAK kinase inhibitors, and do not significantly inhibit the activity of any of the 139 other protein kinases we have investigated (Figures 1 and 2). Consistent with WZ4003 and HTH-01-015 targeting NUAK1 *in vivo*, we observe that these compounds inhibited MYPT1 Ser^445^ phosphorylation as well as cell migration, invasion and proliferation to a similar extent as knock out in MEFs or knock down in U2OS cells of NUAK1. The identification of the A195T mutation that renders NUAK1 ~50-fold resistant to WZ4003 and HTH-01-015 also provides an important approach to validate that biological effects of these compounds are indeed mediated through inhibition of NUAK1 rather than through an off-target effect. Although as a proof of concept, we have shown that overexpression of the NUAK1[A195T] mutant, but not wild-type NUAK1, renders MYPT1 phosphorylation resistant to WZ4003 and HTH-01-015, this approach is not ideal, as the overexpression of NUAK1 has the potential to have an impact on biological processes by inducing non-physiological phosphorylation of cellular proteins. In future work we would recommend that gene-editing technologies be deployed to generate an endogenous NUAK1[A195T] knock-in mutation. Such knock-in cell lines should be rendered greatly resistant to the WZ4003 and HTH-01-015 inhibitors and therefore any effects that these compounds have that is mediated through inhibition of NUAKs should be suppressed by this mutation.

The IC_50_ values of the WZ4003 and HTH-01-015 compounds for inhibiting NUAK1 are in the range 20–100 nM when assayed at 0.1 mM ATP *in vitro*. On the basis of the structures of these compounds, it is likely that they are acting as ATP-competitive inhibitors. As concentrations of ATP in cells are over 20-fold higher than our *in vitro* assays, this is likely to account for why relatively high concentrations of 3–10 μM WZ4003 and HTH-01-015 are required to maximally suppress MYPT1 Ser^445^ phosphorylation *in vivo*. We have devoted considerable effort to generate more potent NUAK1 inhibitors and have indeed identified two analogues of HTH-01-015, namely XMD-17-51 and XMD-18-42, that inhibit NUAK1 with greater potency. However, these compounds suffer from the drawback that they are less selective than WZ4003 and HTH-01-015 and inhibit other kinases implicated in controlling cell growth and proliferation (Figures 3 and 4). XMD-17-51 also partially suppresses several other AMPK family kinases (Figure 3).

WZ4003 inhibits both NUAK1 and NUAK2, whereas HTH-01-015, as well as the more potent XMD-17-51 and XMD-18-42 derivatives, are NUAK1-specific inhibitors. It is currently unknown whether NUAK1 and NUAK2 have redundant roles *in vivo*. Therefore comparing the effects of WZ4003 with NUAK1-selective inhibitors could provide insights into the relative contributions of NUAK isoforms in mediating physiological processes. *In vitro* NUAK1 and NUAK2 are equally efficient at phosphorylating MYPT1 at Ser^445^ and both isoforms interact similarly with the MYPT1–PP1 complex [10]. On the basis of this, it is likely that compounds such as HTH-01-015, which do not inhibit NUAK2, would not suppress MYPT1 phosphorylation to the same extent as the dual NUAK isoform inhibitors. This is indeed what we observe (Figures 5A and 5B, see also Figures 3D and 4D). In future work it would also be interesting to undertake crystallographic analysis of the binding of specific inhibitors to NUAK isoforms in order to elucidate the structural basis for the high specificity and isoform selectivity of WZ4003 and HTH-01-015 compounds. Such knowledge might also help with designing more potent NUAK1 inhibitors that retain high selectivity.

Most importantly, the results of the present study indicate that WZ4003 and HTH-01-015 represent useful chemical probes to dissect the physiological roles of the NUAK kinases. In future studies we would advocate undertaking studies using both structurally diverse WZ4003 and HTH-01-015 inhibitors that could give insight into the relative contributions of NUAK1 and NUAK2 in controlling physiological processes. We also propose that inhibitor-resistant NUAK1[A195T] mutants be deployed to ensure any biological effects arising from NUAK1 inhibitors is indeed desensitized by this mutation. In future studies it will be exciting to establish the effects that WZ4003 and HTH-01-015 have on biological process recently proposed to be controlled by NUAK isoforms, such as Myc-driven tumour cells [19], neuronal polarity [18] and melanoma cell adhesion [11].

## Online data

Supplementary data

Supplementary Movie S1. NUAK1^+/+^ MEFs has a faster migration phenotype compared to NUAK1^-/-^ MEFs

Supplementary Movie S2. Reintroduction of NUAK1^+/+^ into NUAK1^-/-^ MEFs rescued the slow migration phenotype
