# Supplementary material for: Characterization of WZ4003 and HTH-01-015 as selective inhibitors of the LKB1-tumour-suppressor-activated NUAK kinases
Source: Biochem J. 2013 Dec 10;457(Pt 1):215–25. doi: 10.1042/BJ20131152 (PMC3969223; doi:10.1042/BJ20131152)
Supplement: Supplementary data [file bj4570215add.pdf]

BJ ChemBio

Biochem. J. (2014) **457**, 215–225 (Printed in Great Britain) doi:10.1042/BJ20131152

SUPPLEMENTARY ONLINE DATA

# Characterization of WZ4003 and HTH-01-015 as selective inhibitors of the LKB1-tumour-suppressor-activated NUAK kinases

Sourav BANERJEE*, Sara J. BUHLRAGE†‡, Hai-Tsang HUANG†‡, Xianming DENG†‡, Wenjun ZHOU†‡, Jinhua WANG†‡, Ryan TRAYNOR*, Alan R. PRESCOTT§, Dario R. ALESSI*[1] and Nathanael S. GRAY†‡[1]

*MRC Protein Phosphorylation and Ubiquitylation Unit, College of Life Sciences, University of Dundee, Dow Street, Dundee DD1 5EH, U.K.
†Department of Cancer Biology, Dana-Farber Cancer Institute, Boston, MA 02115, U.S.A.
‡Department of Biological Chemistry and Molecular Pharmacology, Harvard Medical School, 250 Longwood Avenue, SGM 628, Boston, MA 02115, U.S.A.
§Division of Cell Signalling and Immunology, College of Life Sciences, University of Dundee, Dow Street, Dundee DD1 5EH, U.K.

## CHEMICAL SYNTHESIS

### Methyl 3-amino-2-naphthoate

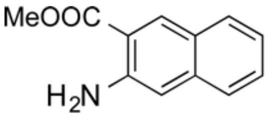

**1**

To a solution of 3-amino-2-naphthoic acid (562 mg, 3.0 mmol, 1.0 eq) in methanol/toluene (1:4, 10 ml) was added 2.0 M trimethylsilyldiazomethane solution in hexane (1.8 ml, 3.6 mmol, 1.2 eq) at 0 °C. The reaction was stirred overnight at room temperature (20 °C). Next day, the reaction was quenched with excess acetic acid until no bubbling was seen. The mixture was directly concentrated *in vacuo*. The residue was purified by silica-gel column chromatography with ethyl acetate and hexane (0–25 % gradient, v/v) to give compound **1** (500 mg, 83 %). $^1$H-NMR (400 MHz, [$^2$H]methanol) $\delta$ 8.46 (s, 1 H), 7.70 (d, *J* 8.2 Hz, 1 H), 7.51 (d, *J* 8.2 Hz, 1 H), 7.37 (ddd, *J* 8.2, 6.8, 1.2 Hz, 1 H), 7.15 (ddd, *J* 8.2, 6.8, 1.2 Hz, 1 H), 7.05 (s, 1 H), 3.93 (s, 3 H). MS (ESI) calculated for $[C_{12}H_{12}NO_2]^+$, 202; found, 202.

### Methyl 3-[(2-chloro-6-methyl-5-nitropyrimidin-4-yl)amino]-2-naphthoate

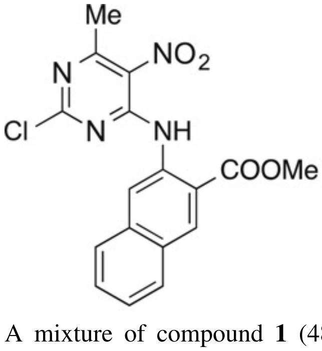

**2**

A mixture of compound **1** (480 mg, 2.4 mmol, 1.0 eq), *N*,*N*-diisopropylethylamine (0.83 ml, 4.8 mmol, 2.0 eq) and 2,4-dichloro-6-methyl-5-nitropyrimidine (0.76 g, 3.6 mmol, 1.5 eq) in 2-propanol (43 ml) was stirred at room temperature overnight. The product crashed out of 2-propanol, and was collected by filtration and dried *in vacuo*. The crude compound **2** (0.79 g, 88 %) was used for the next step without further purification. $^1$H-NMR (400 MHz, [$^2$H]chloroform) $\delta$ 12.04 (s, 1 H), 8.94 (s, 1 H), 8.68 (s, 1 H), 7.89 (d, *J* 8.2 Hz, 2 H), 7.63 (ddd, *J* 8.2, 7.0, 1.2 Hz, 1 H), 7.51 (ddd, *J* 8.2, 7.0, 1.2 Hz, 1 H), 4.05 (s, 3 H), 2.73 (s, 3 H). MS (ESI) calculated for $[C_{17}H_{14}ClN_4O_4]^+$, 373; found, 373.

### 2-chloro-4-methyl-5,13-dihydro-6*H*-naphtho[2,3-*e*]pyrimido[5,4-*b*][1,4]diazepin-6-one

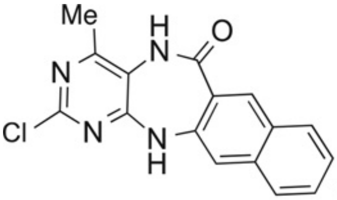

**3**

To a solution of compound **2** (0.79 g, 2.1 mmol, 1.0 eq) in acetic acid (90 ml) was added iron powder (1.7 g, 30.4 mmol, 14.5 eq). The reaction was stirred at 60 °C overnight. After the reaction was complete as monitored by reverse-phase analytical LC–MS, the solvent was removed *in vacuo*. The resulting residue was poured into ice-cold water and stirred, which resulted in a solid precipitate that was collected by filtration, washed with water and air-dried to give compound **3** (0.64 g, 98 %). $^1$H-NMR (400 MHz, [$^2$H]chloroform) $\delta$ 8.63 (s, 1 H), 7.85 (d, *J* 8.2 Hz, 1 H), 7.68 (d, *J* 8.2 Hz, 1 H), 7.54 (ddd, *J* 8.2, 7.0, 1.4 Hz, 1 H), 7.42 (ddd, *J* 8.2, 7.0, 1.2 Hz, 1 H), 7.17 (s, 1 H), 7.15 (s, 1 H), 6.88 (s, 1 H), 2.52 (s, 3 H). MS (ESI) calculated for $[C_{16}H_{12}ClN_4O]^+$, 311; found, 311.

### 2-chloro-4,5,13-trimethyl-5,13-dihydro-6*H*-naphtho[2,3-*e*]pyrimido[5,4-*b*][1,4]diazepin-6-one

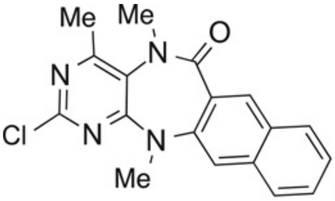

**4**

To a stirred suspension of compound **3** (0.64 g, 2.1 mmol, 1.0 eq) and methyl iodide (0.64 ml, 10.3 mmol, 5.0 eq) in dimethyl acetamide (20.0 ml) was added sodium hydride (300 mg, 60 % suspension in mineral oil, 3.6 eq) at 0 °C. After the reaction was complete as monitored by LC–MS, the solution was poured into ice-cold water, which resulted in a solid precipitate. The precipitate was collected by filtration, washed with water and air-dried to give the crude product. The crude product was purified by silica-gel column chromatography with ethyl acetate and hexane (0–80 % gradient, v/v) to give compound **4** (67 mg, 10 %). $^1$H-NMR (400 MHz, [$^2$H]methanol) $\delta$ 8.30 (s, 1 H), 7.88 (d, *J* 8.2 Hz, 1 H), 7.84 (d, *J* 8.2 Hz, 1 H), 7.61 (s, 1 H), 7.54 (ddd, *J* 8.2, 7.0,

[1] Correspondence may be addressed to either of these authors (email d.r.alessi@dundee.ac.uk or Nathanael_Gray@dfci.harvard.edu).

© 2014 The Author(s)
The author(s) has paid for this article to be freely available under the terms of the Creative Commons Attribution Licence (CC-BY) (http://creativecommons.org/licenses/by/3.0/) which permits unrestricted use, distribution and reproduction in any medium, provided the original work is properly cited.

Biochemical Journal www.biochemj.org

1.2 Hz, 1 H), 7.45 (ddd, *J* 8.2, 7.0, 1.2 Hz, 1 H), 3.51 (s, 3 H), 3.37 (s, 3 H), 2.48 (s, 3 H). MS (ESI) calculated for $[C_{18}H_{16}ClN_4O]^+$, 339; found, 339.

### 4,5,13-trimethyl-2-{[1-(piperidin-4-yl)-1*H*-pyrazol-4-yl]amino}-5,13-dihydro-6*H*-naphtho[2,3-*e*]pyrimido[5,4-*b*][1,4]diazepin-6-one

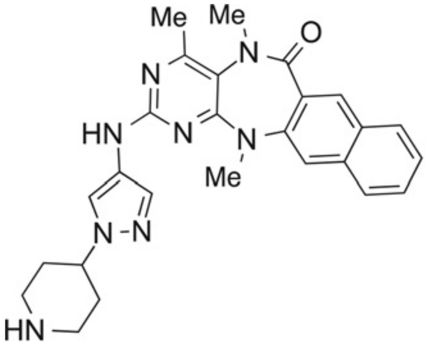


**5, HTH-01-015**

A mixture of compund **4** (34 mg, 0.1 mmol, 1.0 eq), t-butyl 4-(4-amino-1*H*-pyrazol-1-yl)piperidine-1-carboxylate (27 mg, 0.1 mmol, 1.0 eq), X-Phos (8.6 mg, 20 %), tris(dibenzylideneacetone)dipalladium(0) (11 mg, 10 %) and potassium carbonate (41.5 mg, 0.3 mmol) in 1.2 ml of t-butyl alcohol was heated at 85 °C in a sealed tube for 3.5 h. The reaction was then filtered through celite and eluted with dichloromethane. The dichloromethane was removed *in vacuo*. The resulting crude product was stirred with trifluoroacetic acid (0.38 ml, 5 mmol, 50 eq) in dichloromethane (2 ml) at room temperature overnight to afford Boc deprotection. The solvent was removed *in vacuo*. The residue was purified by reverse-phase prep-HPLC using a water (0.05 % trifuloroacetic acid)/methanol (0.05 % trifluoroacetic acid) gradient to afford the title compound HTH-01-015 as a trifluoroacetic acid salt (18 mg, yield 31 %). $^1$H-NMR (400 MHz, DMSO-$d_6$) $\delta$ 9.72–9.40 (br, 1 H), 8.74–8.61 (br, 1 H), 8.54–8.37 (br, 1 H), 8.29 (s, 1 H), 7.97 (d, *J* 8.2 Hz, 1 H), 7.92 (s, 1 H), 7.88 (d, *J* 8.2 Hz, 1 H), 7.67 (s, 1 H), 7.60 (s, 1 H), 7.56 (ddd, *J* 8.2, 7.0, 1.2 Hz, 1 H), 7.46 (ddd, *J* 8.2, 7.0, 1.2 Hz, 1 H), 4.54–4.41 (br, 1 H), 3.54–3.38 (br, 5 H), 3.27 (s, 3 H), 3.17–3.02 (br, 2 H), 2.33 (s, 3 H), 2.26–2.04 (br, 4 H). MS (ESI) calculated for $[C_{26}H_{29}N_8O^+]^+$, 469; found, 469.

### XMD18-42 and XMD17-51

XMD18-42 and XMD17-51 were synthesized following similar strategies as shown in Scheme 1.

### 5,13-dimethyl-2-{[1-(piperidin-4-yl)-1*H*-pyrazol-4-yl]amino}-5,13-dihydro-6*H*-naphtho[2,3-*e*]pyrimido[5,4-*b*][1,4]diazepin-6-one

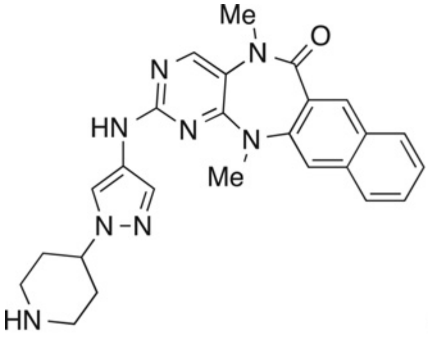


**XMD18-42**

$^1$H-NMR (400 MHz, DMSO-$d_6$) $\delta$ 9.77–9.44 (br, 1 H), 9.18–8.96 (br, 2 H), 8.39 (s, 1 H), 8.33 (s, 1 H), 7.98 (d, *J* 8.2 Hz, 1 H), 7.95 (s, 1 H), 7.91 (d, *J* 8.2 Hz, 1 H), 7.75 (s, 1 H), 7.60 (s, 1 H), 7.57 (t, *J* 8.2 Hz, 1 H), 7.46 (t, *J* 8.2 Hz, 1 H), 4.56–4.43 (br, 1 H), 3.55–3.47 (br, 3 H), 3.45 (s, 3 H), 3.43–3.34 (br, 2 H), 3.13–2.99 (br, 2 H), 2.27–2.09 (br, 4 H). MS (ESI) calculated for $[C_{25}H_{27}N_8O^+]^+$, 455; found, 455.

### 5,11-dimethyl-2-{[1-(piperidin-4-yl)-1*H*-pyrazol-4-yl]amino}-5,11-dihydro-6*H*-benzo[*e*]pyrimido[5,4-*b*][1,4]diazepin-6-one

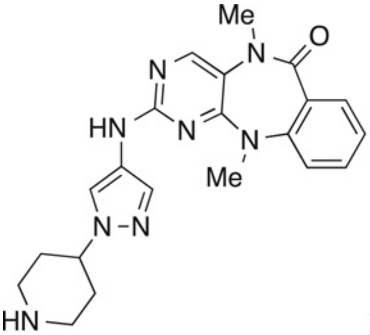


**XMD17-51**

$^1$H-NMR (400 MHz, DMSO-$d_6$) $\delta$ 9.77–9.60 (br, 1 H), 9.16–8.94 (br, 2 H), 8.35 (s, 1 H), 7.93 (s, 1 H), 7.68 (dd, *J* 7.9, 1.8 Hz, 1 H), 7.58 (s, 1 H), 7.51 (td, *J* 7.9, 1.8 Hz, 1 H), 7.28 (d, *J* 7.9 Hz, 1 H), 7.18 (t, *J* 7.9 Hz, 1 H), 4.52–4.44 (br, 1 H), 3.42–3.34 (br, 5 H), 3.38 (s, 3 H), 3.10–3.00 (br, 2 H), 2.21–2.12 (br, 4 H). MS (ESI) calculated for $[C_{21}H_{25}N_8O^+]^+$, 405; found, 405.

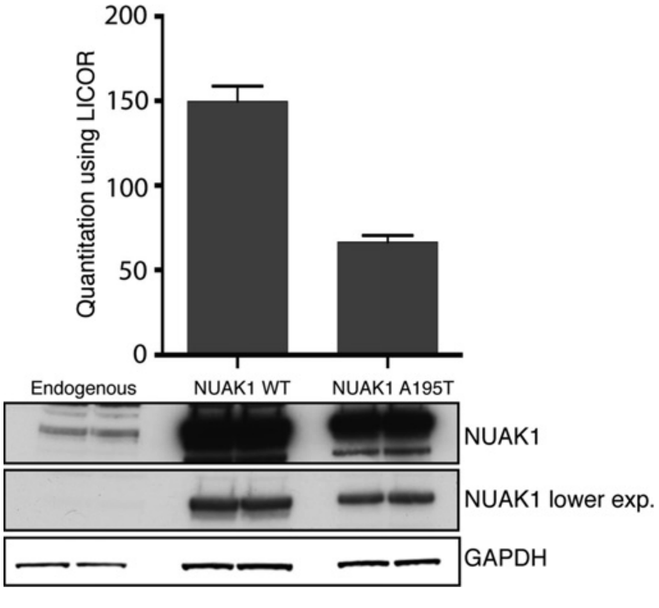


**Figure S1 LI-COR quantification of the overexpression of wild-type (WT) and inhibitor-resistant NUAK1[A195T] mutant**

The Western blot signals for endogenous and overexpressed NUAK1 in HEK-293 cells were quantified using LI-COR Odyssey technology. GAPDH (glyceraldehyde-3-phosphate dehydrogenase) was used as a loading control. The background signal was subtracted and then the band intensity of overexpressed NUAK1 was divided by the signal for the endogenous NUAK1 protein (in duplicate). Data are represented relative to the expression levels of the endogenous protein. lower exp., lower exposure.

© 2014 The Author(s)
The author(s) has paid for this article to be freely available under the terms of the Creative Commons Attribution Licence (CC-BY) (http://creativecommons.org/licenses/by/3.0/) which permits unrestricted use, distribution and reproduction in any medium, provided the original work is properly cited.

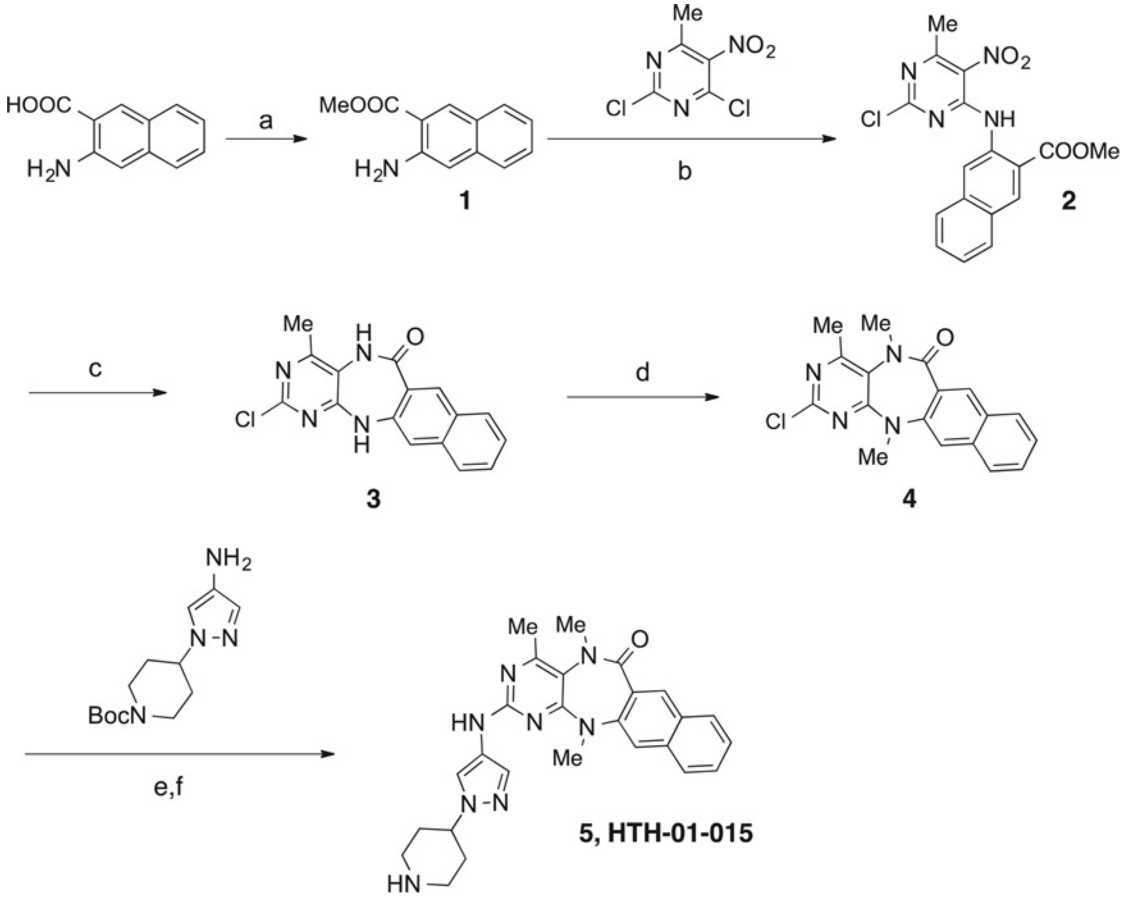


**Scheme S1 Synthesis of 4,5,13-trimethyl-2-{[1-(piperidin-4-yl)-1*H*-pyrazol-4-yl]amino}-5,13-dihydro-6*H*-naphtho[2,3-*e*]pyrimido[5,4-*b*][1,4]diazepin-6-one (HTH-01-015)**

Reagents and conditions: (a) trimethylsilyldiazomethane (1.2 eq.), methanol/toluene (1:4), 0°C; (b) *N*,*N*-diisopropylethylamine (2.0 eq), 2-propanol; (c) Fe (14.5 eq.), acetic acid, 60°C; (d) methyl iodide (5.0 eq.), sodium hydride (3.6 eq.), dimethyl acetamide, 0°C; (e) X-Phos (20% mol), tris(dibenzylideneacetone)dipalladium(0) (10% mol), potassium carbonate (3.0 eq.), t-butyl alcohol, 85°C; (f) trifluoroacetic acid (50 eq), dichloromethane.

© 2014 The Author(s)
The author(s) has paid for this article to be freely available under the terms of the Creative Commons Attribution Licence (CC-BY) (http://creativecommons.org/licenses/by/3.0/) which permits unrestricted use, distribution and reproduction in any medium, provided the original work is properly cited.

**Table S1 Effect of the NUAK inhibitors upon the activity of 140 protein kinases**

Results are presented as the percentage of kinase activity in DMSO control reactions. Protein kinases were assayed *in vitro* with 0.1 or 1 $\mu$M of the inhibitors as described previously [1], and the results are means $\pm$ S.D. for triplicate reactions. *Indicates AMPK-related kinase family members. Abbreviations are as follows: ABL, Abelson tyrosine-protein kinase 1; AMPK, AMP-activated protein kinase; ASK, apoptosis signal-regulating kinase; BRK, breast tumour kinase; BRSK, brain-specific kinase; BTK, Bruton"s tyrosine kinase; CaMK, calmodulin-dependent kinase; CaMKK, CaMK kinase; CDK, cyclin-dependent kinase; CHK, checkpoint kinase; CK, casein kinase; CLK, CDC-like kinase; CSK, C-terminal Src kinase; DAPK, death-associated protein kinase; DDR, discoidin domain receptor; DYRK, dual-specificity tyrosine-phosphorylated and regulated kinase; EF2K, elongation-factor-2 kinase; EIF2AK, eukaryotic translation initiation factor 2-alpha kinase; EPH, ephrin; ERK, extracellular signal-regulated kinase; FGF-R, fibroblast growth factor receptor; GCK, germinal centre kinase; GSK, glycogen synthase kinase; HER, human epidermal growth factor receptor; HIPK, homeodomain-interacting protein kinase; IGF1R, IGF1 receptor; IKK, inhibitory $\kappa$B kinase; IR, insulin receptor; IRAK, interleukin-1 receptor-associated kinase; IRR, insulin-related receptor; JAK, Janus kinase; JNK, c-Jun N-terminal kinase; Lck, lymphocyte cell-specific protein tyrosine kinase; LKB1, liver kinase B1; MAPK, mitogen-activated protein kinase; MAPKAP-K, MAPK-activated protein kinase; MARK, microtubule-affinity-regulating kinase; MEKK, MAP kinase kinase kinase; MELK, maternal embryonic leucine-zipper kinase; MINK, misshapen/NIK-related kinase; MKK, MAPK kinase; MLK, mixed lineage kinase; MNK, MAPK-integrating protein kinase; MPSK, myristoylated and palmitoylated serine/threonine-protein kinase; MSK, mitogen- and stress-activated protein kinase; MST, mammalian homologue Ste20-like kinase; NEK, NIMA (never in mitosis in *Aspergillus nidulans*)-related kinase; NUAK, novel (NUA) family SnF1-like kinase; OSR, oxidative stress-responsive kinase; PAK, p21-activated protein kinase; PDGFRA, platelet-derived growth factor receptor-$\alpha$; PDK, phosphoinositide-dependent kinase; PHK, phosphorylase kinase; PIM, provirus integration site for Moloney murine leukaemia virus; PINK (insect homologue), PTEN-induced kinase; PKA, cAMP-dependent protein kinase; PKB, protein kinase B; PKC, protein kinase C; PKD, protein kinase D; PLK, polo-like kinase; PRAK, p38-regulated activated kinase; PRK, protein kinase C-related kinase; RIPK, receptor-interacting protein kinase; ROCK, Rho-dependent protein kinase; RSK, ribosomal S6 kinase; S6K, p70 ribosomal S6 kinase; SGK, serum- and glucocorticoid-induced protein kinase; SIK, salt-induced kinase; smMLCK, smooth muscle myosin light-chain kinase; SRPK, serine/arginine protein kinase; STK, serine/threonine kinase; SYK, spleen tyrosine kinase; TAK, TGF$\beta$-activated kinase; TAO, thousand and one amino acid; TBK1, TANK-binding kinase 1; TESK, testis-specific protein kinase; TGFBR, TGF$\beta$ receptor; TIE, tyrosine-protein kinase receptor; TLK, tousled-like kinase; TrkA, tropomyocin receptor kinase; TSSK, testis-specific serine/threonine-protein kinase; TTBK, tau-tubulin kinase; ULK, Unc-51-like kinase; VEGFR, vascular endothelial growth factor receptor; WNK, with no lysine; YES1, Yamaguchi sarcoma viral oncogene homologue 1; ZAP, $\zeta$-chain-associated protein.

| Kinase | HTH-01-015 | WZ4003 |
|---|---|---|
| **NUAK1*** | **11 ± 0** | **6 ± 0** |
| AMPK* | 127 ± 9 | 89 ± 9 |
| MARK1* | 106 ± 4 | 76 ± 9 |
| MARK2* | 99 ± 4 | 85 ± 14 |
| MARK3* | 102 ± 1 | 51 ± 2 |
| MARK4* | 75 ± 2 | 79 ± 3 |
| BRSK1* | 130 ± 32 | 91 ± 1 |
| BRSK2* | 97 ± 3 | 75 ± 22 |
| MELK* | 99 ± 9 | 70 ± 2 |
| SIK2* | 112 ± 16 | 70 ± 0 |
| SIK3* | 109 ± 32 | 102 ± 9 |
| LKB1 | 98 ± 10 | 87 ± 0 |
| MKK1 | 113 ± 28 | 98 ± 14 |
| MKK2 | 111 ± 10 | 98 ± 4 |
| MKK6 | 87 ± 1 | 101 ± 4 |
| ERK1 | 83 ± 1 | 94 ± 2 |
| ERK2 | 125 ± 6 | 107 ± 6 |
| ERK5 | 121 ± 4 | 79 ± 2 |
| JNK1 | 100 ± 2 | 88 ± 4 |
| JNK2 | 130 ± 9 | 97 ± 3 |
| JNK3 | 101 ± 8 | 70 ± 2 |
| p38$\alpha$ MAPK | 118 ± 4 | 95 ± 4 |
| p38$\beta$ MAPK | 112 ± 3 | 104 ± 6 |
| p38$\gamma$ MAPK | 100 ± 0 | 91 ± 0 |
| p38$\delta$ MAPK | 113 ± 1 | 63 ± 10 |
| ERK8 | 98 ± 7 | 90 ± 9 |
| RSK1 | 93 ± 3 | 67 ± 5 |

**Table S1 Continued**

| Kinase | HTH-01-015 | WZ4003 |
|---|---|---|
| RSK2 | 106 ± 15 | 76 ± 1 |
| PDK1 | 102 ± 5 | 104 ± 10 |
| PKB$\alpha$ | 87 ± 15 | 98 ± 3 |
| PKB$\beta$ | 104 ± 2 | 114 ± 34 |
| SGK1 | 97 ± 2 | 99 ± 7 |
| S6K1 | 85 ± 3 | 83 ± 14 |
| PKA | 101 ± 0 | 84 ± 18 |
| ROCK 2 | 110 ± 14 | 80 ± 2 |
| PRK2 | 112 ± 8 | 99 ± 20 |
| PKC$\alpha$ | 117 ± 4 | 77 ± 8 |
| PKC$\gamma$ | 107 ± 0 | 103 ± 15 |
| PKC$\zeta$ | 98 ± 13 | 86 ± 15 |
| PKD1 | 94 ± 8 | 57 ± 5 |
| STK33 | 93 ± 2 | 33 ± 7 |
| MSK1 | 111 ± 2 | 90 ± 3 |
| MNK1 | 102 ± 9 | 104 ± 8 |
| MNK2 | 106 ± 0 | 84 ± 16 |
| MAPKAP-K2 | 118 ± 9 | 82 ± 10 |
| MAPKAP-K3 | 96 ± 20 | 91 ± 5 |
| PRAK | 105 ± 10 | 94 ± 0 |
| CAMKK$\beta$ | 87 ± 12 | 44 ± 2 |
| CAMK1 | 92 ± 0 | 81 ± 1 |
| SmMLCK | 77 ± 8 | 78 ± 2 |
| PHK | 104 ± 24 | 54 ± 11 |
| DAPK1 | 100 ± 5 | 93 ± 12 |
| CHK1 | 106 ± 6 | 58 ± 1 |
| CHK2 | 105 ± 11 | 40 ± 0 |
| GSK3$\beta$ | 119 ± 13 | 88 ± 0 |
| CDK2-Cyclin A | 96 ± 3 | 90 ± 32 |
| CDK9-Cyc T1 | 68 ± 1 | 89 ± 2 |
| PLK1 | 98 ± 5 | 84 ± 9 |
| Aurora A | 117 ± 1 | 75 ± 11 |
| Aurora B | 97 ± 13 | 72 ± 8 |
| TLK1 | 106 ± 10 | 91 ± 7 |
| TSSK1 | 95 ± 7 | 58 ± 11 |
| CK1$\gamma$2 | 109 ± 11 | 102 ± 4 |
| CK1$\delta$ | 94 ± 5 | 97 ± 5 |
| CK2 | 120 ± 11 | 76 ± 0 |
| TTBK1 | 124 ± 19 | 78 ± 21 |
| TTBK2 | 102 ± 4 | 89 ± 3 |
| DYRK1A | 89 ± 4 | 102 ± 2 |
| DYRK2 | 113 ± 7 | 66 ± 8 |
| DYRK3 | 98 ± 0 | 71 ± 9 |
| NEK2$\alpha$ | 103 ± 2 | 110 ± 3 |
| NEK6 | 99 ± 13 | 79 ± 13 |
| IKK$\beta$ | 81 ± 9 | 72 ± 0 |
| IKK$\varepsilon$ | 85 ± 1 | 104 ± 0 |
| TBK1 | 112 ± 8 | 84 ± 5 |
| PIM1 | 92 ± 4 | 79 ± 7 |
| PIM2 | 106 ± 6 | 101 ± 21 |
| PIM3 | 97 ± 2 | 98 ± 1 |
| SRPK1 | 103 ± 7 | 94 ± 1 |
| EF2K | 103 ± 8 | 91 ± 2 |
| EIF2AK3 | 99 ± 13 | 76 ± 17 |
| HIPK1 | 107 ± 7 | 103 ± 20 |
| HIPK2 | 107 ± 6 | 86 ± 19 |
| HIPK3 | 96 ± 13 | 91 ± 1 |
| CLK2 | 54 ± 1 | 65 ± 7 |
| PAK2 | 125 ± 13 | 92 ± 6 |
| PAK4 | 110 ± 8 | 69 ± 6 |
| PAK5 | 95 ± 5 | 89 ± 5 |
| PAK6 | 95 ± 2 | 104 ± 1 |
| MST2 | 118 ± 1 | 91 ± 2 |
| MST3 | 111 ± 3 | 93 ± 8 |
| MST4 | 119 ± 7 | 101 ± 0 |
| GCK | 126 ± 3 | 96 ± 4 |
| MAP4K3 | 127 ± 5 | 111 ± 15 |
| MAP4K5 | 106 ± 4 | 108 ± 2 |
| MINK1 | 101 ± 1 | 120 ± 5 |
| MEKK1 | 110 ± 14 | 91 ± 1 |

© 2014 The Author(s)
The author(s) has paid for this article to be freely available under the terms of the Creative Commons Attribution Licence (CC-BY) (http://creativecommons.org/licenses/by/3.0/) which permits unrestricted use, distribution and reproduction in any medium, provided the original work is properly cited.

**Table S1 Continued**

| Kinase | HTH-01-015 | WZ4003 |
|---|---|---|
| MLK1 | 102 ± 18 | 69 ± 0 |
| MLK3 | 94 ± 7 | 73 ± 8 |
| TESK1 | 106 ± 8 | 98 ± 12 |
| TAO1 | 123 ± 10 | 101 ± 7 |
| ASK1 | 105 ± 2 | 107 ± 7 |
| TAK1 | 109 ± 7 | 85 ± 10 |
| IRAK1 | 103 ± 2 | 88 ± 2 |
| IRAK4 | 103 ± 11 | 92 ± 3 |
| RIPK2 | 88 ± 4 | 105 ± 0 |
| OSR1 | 107 ± 4 | 89 ± 1 |
| TTK | 118 ± 3 | 54 ± 17 |
| MPSK1 | 103 ± 5 | 107 ± 9 |
| WNK1 | 99 ± 7 | 95 ± 7 |
| ULK1 | 132 ± 19 | 52 ± 2 |
| ULK2 | 106 ± 13 | 37 ± 2 |
| TGFBR1 | 102 ± 13 | 102 ± 7 |
| Src | 76 ± 8 | 109 ± 10 |
| Lck | 90 ± 3 | 99 ± 3 |
| CSK | 103 ± 5 | 105 ± 0 |
| YES1 | 76 ± 7 | 89 ± 5 |
| ABL | 110 ± 3 | 104 ± 9 |
| BTK | 109 ± 4 | 114 ± 14 |
| JAK2 | 101 ± 11 | 65 ± 2 |
| SYK | 125 ± 5 | 93 ± 4 |
| ZAP70 | 103 ± 2 | 89 ± 6 |
| TIE2 | 84 ± 12 | 94 ± 0 |
| BRK | 96 ± 0 | 79 ± 0 |
| EPH-A2 | 84 ± 1 | 91 ± 13 |
| EPH-A4 | 94 ± 8 | 106 ± 7 |
| EPH-B1 | 97 ± 15 | 98 ± 3 |
| EPH-B2 | 97 ± 15 | 107 ± 13 |
| EPH-B3 | 120 ± 1 | 113 ± 39 |
| EPH-B4 | 102 ± 4 | 83 ± 2 |
| FGF-R1 | 105 ± 11 | 66 ± 2 |
| HER4 | 102 ± 5 | 76 ± 22 |
| IGF-1R | 133 ± 26 | 33 ± 4 |
| IR | 105 ± 0 | 92 ± 12 |
| IRR | 106 ± 6 | 88 ± 2 |
| TrkA | 82 ± 1 | 85 ± 2 |
| DDR2 | 91 ± 5 | 86 ± 21 |
| VEG-FR | 97 ± 12 | 74 ± 21 |
| PDGFRA | 109 ± 5 | 96 ± 2 |
| PINK | 99 ± 1 | 95 ± 7 |

## REFERENCE

1 Najafov, A., Sommer, E. M., Axten, J. M., Deyoung, M. P. and Alessi, D. R. (2011) Characterization of GSK2334470, a novel and highly specific inhibitor of PDK1. Biochem. J. **433**, 357–369

Received 27 August 2013/29 October 2013; accepted 30 October 2013
Published as BJ Immediate Publication 30 October 2013, doi:10.1042/BJ20131152

© 2014 The Author(s)
The author(s) has paid for this article to be freely available under the terms of the Creative Commons Attribution Licence (CC-BY) (http://creativecommons.org/licenses/by/3.0/) which permits unrestricted use, distribution and reproduction in any medium, provided the original work is properly cited.
